# Larger muscle fibers and fiber bundles manifest smaller elastic modulus in paraspinal muscles of rats and humans

**DOI:** 10.1038/s41598-021-97895-z

**Published:** 2021-09-17

**Authors:** Masoud Malakoutian, Marine Theret, Shun Yamamoto, Iraj Dehghan-Hamani, Michael Lee, John Street, Fabio Rossi, Stephen H. M. Brown, Thomas R. Oxland

**Affiliations:** 1 Department of Mechanical Engineering, University of British Columbia, Vancouver, Canada; 2 ICORD, University of British Columbia, Vancouver, Canada; 3 Biomedical Research Center, Department of Medical Genetics and School of Biomedical Engineering, University of British Columbia, Vancouver, Canada; 4 Department of Orthopaedic Surgery, Jikei University Graduate School of Medicine, Tokyo, Japan; 5 Department of Orthopaedics, University of British Columbia, Vancouver, Canada; 6 Department of Human Health and Nutritional Sciences, University of Guelph, Guelph, Canada; 7 UBC Department of Orthopaedics, ICORD, Blusson Spinal Cord Centre, 3rd Floor-818 West 10th Avenue, Vancouver, BC V5Z 1M9 Canada

**Keywords:** Biomedical engineering, Mechanical engineering, Tissues, Skeletal muscle

## Abstract

The passive elastic modulus of muscle fiber appears to be size-dependent. The objectives of this study were to determine whether this size effect was evident in the mechanical testing of muscle fiber bundles and to examine whether the muscle fiber bundle cross-section is circular. Muscle fibers and fiber bundles were extracted from lumbar spine multifidus and longissimus of three cohorts: group one (G1) and two (G2) included 13 (330 ± 14 g) and 6 (452 ± 28 g) rats, while Group 3 (G3) comprised 9 degenerative spine patients. A minimum of six muscle fibers and six muscle fiber bundles from each muscle underwent cumulative stretches, each of 10% strain followed by 4 minutes relaxation. For all specimens, top and side diameters were measured. Elastic modulus was calculated as tangent at 30% strain from the stress–strain curve. Linear correlations between the sample cross sectional area (CSA) and elastic moduli in each group were performed. The correlations showed that increasing specimen CSA resulted in lower elastic modulus for both rats and humans, muscle fibers and fiber bundles. The median ratio of major to minor axis exceeded 1.0 for all groups, ranging between 1.15–1.29 for fibers and 1.27–1.44 for bundles. The lower elastic moduli with increasing size can be explained by relatively less collagenous extracellular matrix in the large fiber bundles. Future studies of passive property measurement should aim for consistent bundle sizes and measuring diameters of two orthogonal axes of the muscle specimens.

## Introduction

Passive stiffness is an important property of skeletal muscles. In particular, the passive elastic modulus of a muscle has a direct impact on the amount of force generated in resistance to lengthening and hence is critical in biomechanical modeling. Elastic modulus also has a pivotal role in cell mechanics and affects function, differentiation, and the proliferation of muscle cells. For example, myofibroblasts have been shown to contract and secrete more extracellular matrix on a stiffer substrate^1^, while their differentiation and function are impeded below certain elastic modulus thresholds^2–4^.

Interestingly, many studies have reported that muscles in individuals with orthopedic pathologies manifest significantly different elastic moduli than muscles of healthy subjects, in both humans^5–9^ and animals^10–12^. The differences were evident both at the cellular level (i.e. for a single muscle fiber)^5,6,9,11^ and the tissue level (i.e. for a bundle of muscle fibers ensheathed in their connective tissue), with most differences existing at the tissue level^6,7,10,12,13^. In some cases, muscle fiber bundles have been reported to be up to four times stiffer in normal population than compared to the patients with pathology (spasticity)^6^. These observations highlight the importance of elastic modulus in muscle physiology, pathology, and biomechanics and encourage more research to be conducted for better understanding and addressing musculoskeletal conditions.

Researchers characterize the passive stiffness of muscle fibers and fiber bundles with a uniaxial stretch test to determine the elastic modulus. They test fibers and fiber bundles of different sizes and normalize for cross-sectional area (CSA) assuming that this eliminates the effect of size (i.e. CSA). The size of muscle fiber bundles tested to determine elastic modulus varies from 3 to 30 fibers in the literature^6,10,11,14–16^. Whether the effect of size on elastic modulus disappears after normalizing for CSA is unknown. A recent study suggested that elastic modulus does depend on the size of the muscle fiber^17^. This question has not been solved for bundles of muscle fibers.

Another important point regarding the measurement of elastic modulus is that most studies of single muscle fibers or bundles of muscle fibers assumed a cylindrical shape and only measured diameter of those specimens in one plane^14,15,18–21^. Other investigators believe measurement of diameters in two different planes (i.e. both top and side views) is necessary^16,22^. Blinks^23^ examined 16 frog fibers and found ~ 20% difference in the calculated CSA when using only one diameter versus two. For humans and rodents, whether the assumption of a cylindrical shape is acceptable and to what extent it influences the measured CSA of a muscle fiber or a bundle of muscle fibers remains unknown.

Therefore, the objectives of this study were (1) to explore whether the size of a single muscle fiber or a bundle of muscle fibers has an influence on its measured elastic modulus; and (2) to examine whether the assumption of a circular shape for the cross section of a muscle fiber or a bundle of fibers is valid. We include samples from two types of paraspinal muscle, multifidus and longissimus, in both rats and humans.

## Methods

### Study groups

Under anesthesia, fresh muscle biopsies from the lumbar paraspinal muscles of two groups of rats and one group of humans were collected. Group 1 consisted of 13 male Sprague Dawley rats ~ 8 weeks old (330 ± 14 g) that had undergone no experimental procedure. Group 2 included six Sprague Dawley rats ~ 21 weeks old (452 ± 28 g) that had undergone a sham surgery with a small midline incision on their thoracolumbar fascia ~ 13 weeks before collecting biopsies. Group 3 was comprised of nine patients with degenerative spinal deformity undergoing a spinal surgery (Table S1). The animal study was ethically approved by the University of British Columbia animal care committee and was conducted in compliance with the relevant guidelines and regulations including ARRIVE guidelines, for which the muscle biopsies were collected when the rats were anesthetized using isoflurane. The human study was approved by Vancouver Coastal Health Research Institute (VCHRI) and the Clinical Research Ethics Board (CREB) of the University of British Columbia, Vancouver, Canada. Informed consents were obtained from all patients and all methods including biopsy collection were carried out in accordance with relevant guidelines and regulations.

A minimum of two biopsies from multifidus and two biopsies from longissimus were collected from each rat/patient. The biopsies were taken from L1, L3, and L5 in rats and L4/L5 in humans. All fresh biopsies were treated similarly: they were transferred to a physiological storage solution immediately after harvest, and after 24 h incubation at 4 °C were stored at − 20 °C for a minimum of 24 h. Mechanical properties of muscle tissues stored in this way are expected to remain stable for up to 3 months^24,25^. Later, the biopsies were transferred to a cold relaxing solution under a dissecting microscope to extract single fibers and bundles of fibers for mechanical testing.

**Figure 1:**
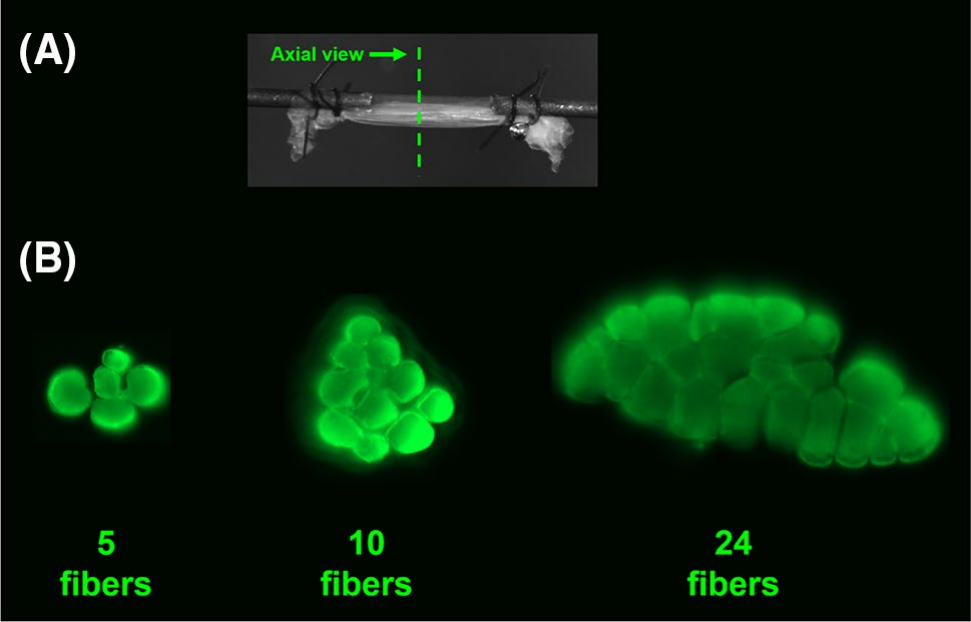
Muscle fiber bundle specimen for mechanical testing; (**A**) The specimens were mounted onto two collinear pins and secured to them via suture loops. (**B**) Axial view of three tested muscle fiber bundles. Out of six bundles extracted from a biopsy, two would typically be small (~ 7 fibers), two medium (~ 14 fibers), and two large (~ 21 fibers).

From each biopsy, two to three single fibers and two to six bundles of fibers were extracted for measurement of their geometric dimensions and elastic modulus. While the maintenance of a balance in the size range of extracted bundles of fibers was attempted, more emphasis was placed on taking extreme care to maintain the integrity of the bundles during extraction. For instance, out of six bundles extracted from a biopsy, two would typically be small (~ 7 fibers), two medium (~ 14 fibers), and two large (~ 21 fibers; Fig. 1). Based on previous work showing no difference between the spinal levels^26^, all samples belonging to the same study group and the same muscle group were put into one pool for data analysis. For example, for study group 1 (G1) four types of samples were pooled: G1 Multifidus Fibers, G1 Longissimus Fibers, G1 Multifidus Bundles, and G1 Longissimus Bundles.

### Mechanical testing

The same methodology as utilized by Malakoutian et al.^26^ was followed for mechanical testing and measurement of elastic modulus. Briefly, each fiber/fiber bundle was immersed into a bath of physiological relaxing solution and was secured at its ends to two collinear pins with outer diameter of 0.15 mm (No. 26002-15, Fine Science Tools, BC, Canada). One pin was connected to a highly sensitive force transducer (400A, Aurora Scientific, Ontario, Canada) and the other pin was attached to a length controller (CRK523PMAP, Oriental Motor, USA). The fiber or fiber bundle was lengthened until it reached its slack sarcomere length. Slack sarcomere length was determined as the first detectable rise in passive force beyond the noise level of the force transducer; this corresponded to approximately 5 µN for fibers and 50 µN for fiber bundles. Using a crosshair reticle inside the eye piece of a stereomicroscope, top and side diameters were measured with a resolution of 1 micron at three different points along the specimen. The average of the readings for the top and side diameters at those three points were used to calculate the CSA of each specimen. From the slack length, each specimen underwent cumulative (four to eight) stretches, each of 10% strain at a rate of 10% strain per second followed by 4 minutes relaxation^16^. The force reading at the end of each increment was divided by the CSA to obtain the engineering stress. The specimens were transilluminated by a single mode fiber-coupled diode laser (660 nm) and the resulting diffraction pattern was scanned by a photodiode array to measure (with a resolution of ~ 10 nm) the sarcomere length of the specimen prior to any stretches and at the end of each increment. This enabled calculation of the strain after each increment. Elastic modulus was calculated as tangent at 30% strain from the stress–strain curve. All tests were performed within 3 weeks after collection of muscle biopsies.

### Statistical analysis

For each pool of samples (e.g. G3 Longissimus Bundles), a linear regression approach was taken to find a correlation between the CSA (independent variable) of samples and their elastic moduli (dependent variable). The slopes, intercept, and correlation of determination were calculated for each analysis. Also, for each pool of samples the ratios of major over minor axis diameters were calculated and the median and inter quartile range (IQR) were determined. The results were contrasted against the theoretical median of 1 (for cylindrical shape assumption) using Wilcoxon signed rank test.

## Results

In total, 391 fibers (192 in G1, 112 in G2, 87 in G3) and 570 bundles of fibers (262 in G1, 137 in G2, and 171 in G3) were tested. Twenty-six outliers in G1, 19 outliers in G2 and 20 outliers in G3 were removed for having modulus values 1.5 times the interquartile range above the third quartile of their group.

**Figure 2:**
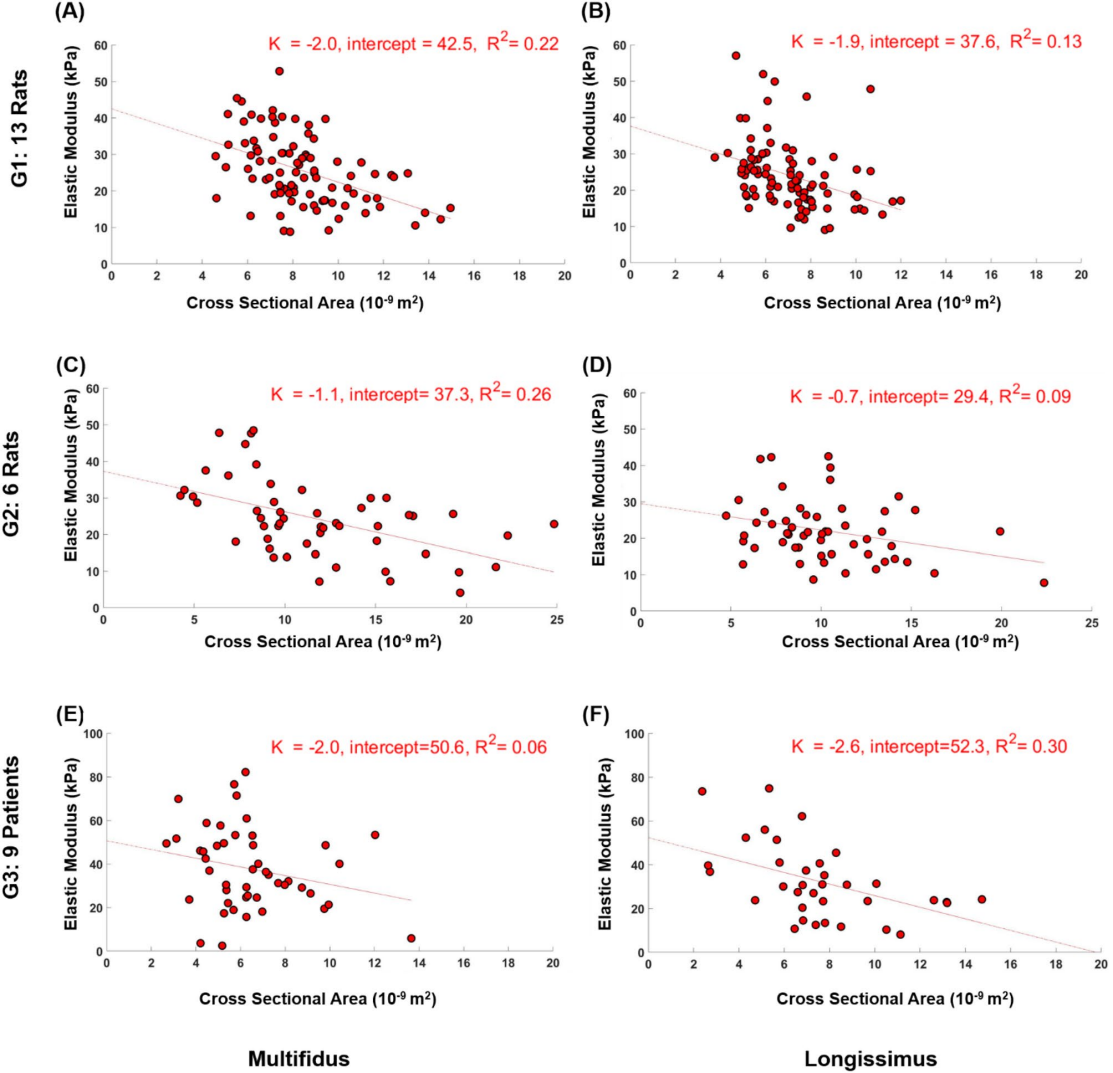
Correlation between cross sectional area and elastic modulus of single fibers from multifidus and longissimus of G1 (**A**,**B**), G2 (**C**,**D**), and G3 (**E**,**F**). All P < 0.026 except for G3 multifidus fibers.

**Figure 3:**
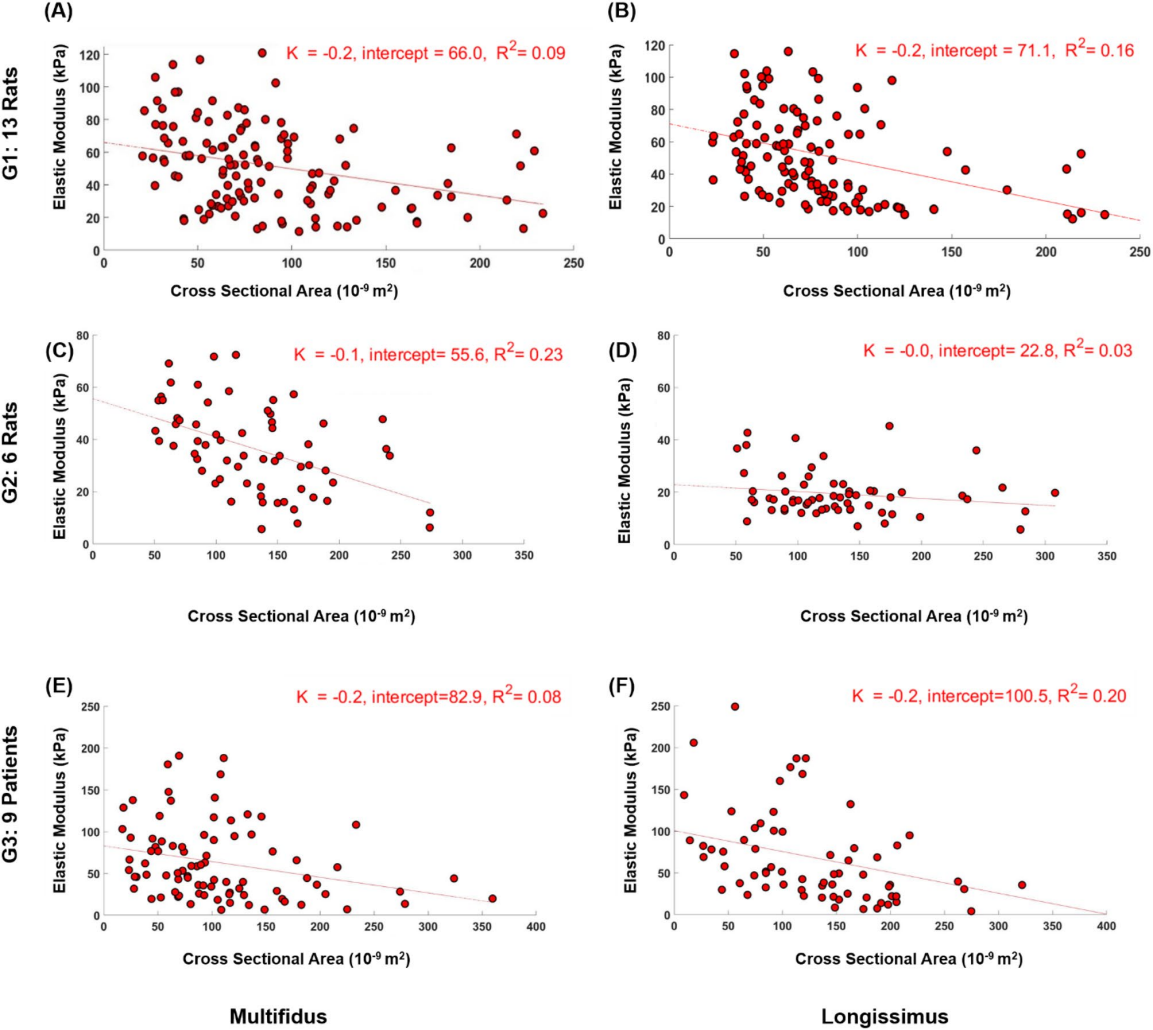
Correlation between cross sectional area and elastic modulus of fiber bundles from multifidus and longissimus of G1 (**A**,**B**), G2 (**C**,**D**), and G3 (**E**,**F**). All P < 0.02 except for G2 longissimus bundles that P = 0.16 (**D**).

All groups manifested a trend of smaller elastic modulus associated with larger fibers and larger bundles of fibers in the paraspinal muscles (Figs. 2 and 3). The correlation of determination ${R}^{2}$ for all groups ranged between 0.06 and 0.30 for muscle fibers (p < 0.05, except for G3 multifidus fibers where p = 0.10; Fig. 2). For the six groups of muscle fiber bundles, the correlation of determination ranged between 0.03 and 0.23 (p < 0.05, except for G2 longissimus bundles where p = 0.16; Fig. 3).

**Figure 4:**
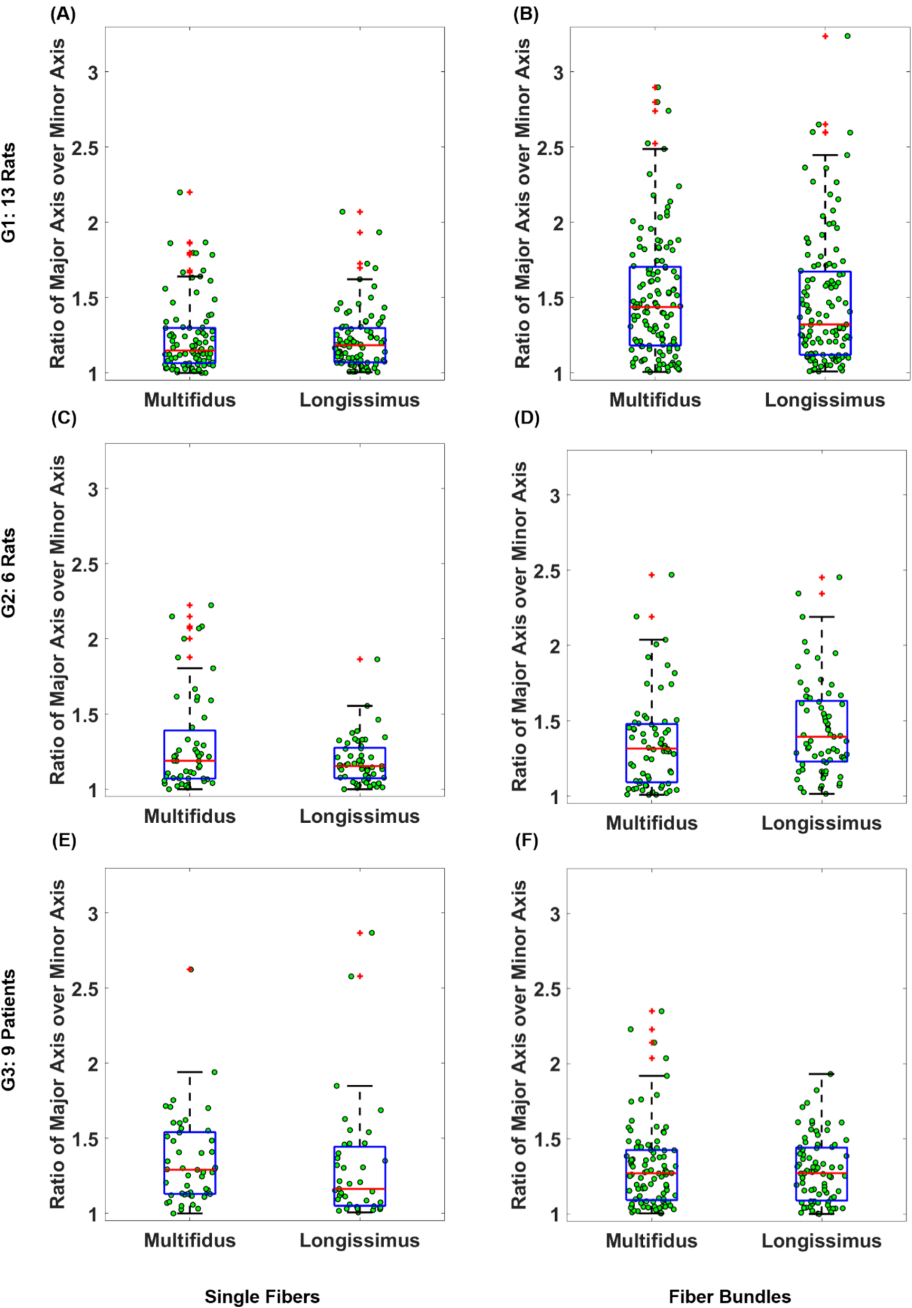
Boxplot representation of ratio of major over minor axes of single fibers and fiber bundles tested in G1 (**A**,**B**), G2 (**C**,**D**), and G3 (**E**,**F**). The red lines represent the medians, the heights of the boxes represent the interquartile ranges and red crosses identify the outliers.

The Wilcoxon signed rank test rejected the cylindrical assumption for the cross section of fibers and fiber bundles (all p < 0.00001). The ratio of the major axis over minor axis had a median (IQR) of 1.15 (0.23), 1.19 (0.32), and 1.29 (0.41) for multifidus fibers of G1, G2, and G3 respectively; and 1.18 (0.23), 1.15 (0.20), and 1.16 (0.39) for longissimus fibers of G1, G2, and G3, respectively (Fig. 4). The range of minor axis diameters for multifidus fibers were from 0.060 to 0.138 mm for G1; 0.049 mm to 0.162 mm for G2, and from 0.034 to 0.110 mm in G3; while for longissimus fibers the range of minor axis diameters were from 0.058 to 0.117 mm for G1, 0.078 mm to 0.133 mm for G2, and 0.048 mm to 0.121 mm for G3.

Bundles of fibers in general had larger ratios compared to fibers. The ratio of the major axis over minor axis had a median (IQR) of 1.44 (0.52), 1.32 (0.39), and 1.27 (0.33) for multifidus fiber bundles of G1, G2, and G3 respectively; and 1.32 (0.55), 1.39 (0.4), and 1.27 (0.35) for longissimus fiber bundles of G1, G2, and G3, respectively (Fig. 4). The range of minor axis diameters for multifidus fiber bundles were from 0.121 to 0.472 mm for G1; 0.208 mm to 0.703 mm for G2, and from 0.126 to 0.622 mm in G3; while for longissimus fibers the range of minor axis diameters were from 0.122 to 0.504 mm for G1, 0.198 mm to 0.578 mm for G2, and 0.078 mm to 0.733 mm for G3.

## Discussion

Muscle passive stiffness provides insight into the biomechanical function, physiology, and health of our musculoskeletal system; thus, its accurate measurement is important. Most studies do not differentiate between bundles of muscle fibers with different sizes. Thus, these studies assume that the elastic modulus (i.e. passive stiffness/CSA) of these muscle specimens is independent of size. The same assumption is made for single fibers. The results of our current study demonstrated that the size matters and in general, larger sizes were associated with lower elastic moduli in fibers and bundles of fibers. Therefore, future studies should consider maintaining consistent bundle sizes for measurement of passive properties especially when comparing different groups against each other.

This same finding was observed for single fibers recently by Noonan et al.^20^ where larger fibers in vastus lateralis of 10 healthy volunteers manifested smaller elastic moduli. They demonstrated that considering a constant thickness and higher elastic modulus for the basement membrane, relative to the modulus of the contractile area of the fiber, results in larger elastic modulus for smaller fibers. The effect is due to the CSA of the basement membrane being proportional to the fiber diameter while the contractile area of a fiber is proportional to its diameter squared. At the fiber bundle level however, no study has examined the effect of size on elastic modulus.

The lower elastic modulus of larger bundles in the current study may arise from their extracellular matrix (ECM) content. It is well established that bundles of fibers have a larger elastic modulus than single fibers due to the high stiffness of ECM^10,19,21,27,28^. Assuming that the ECM and fibers within a bundle are both homogenous, the elastic modulus of a bundle can be calculated following the rule of mixture for composites as:

$${E}_{Bundle}={f}_{ECM}{E}_{ECM}+\left(1-{f}_{ECM}\right){E}_{Fiber}$$

where ${E}_{Bundle}, {E}_{ECM},$ and ${E}_{Fiber}$ are the elastic moduli of the bundle, the extracellular matrix, and fibers, respectively; and ${f}_{ECM}$ denotes the percentage of the extracellular matrix within the bundle. For example, the elastic modulus of a bundle containing 5% ECM (i.e. ${f}_{ECM}=0.05$) with a fiber elastic modulus of 20 kPa and ECM elastic modulus of 1 MPa^28^ is calculated as 69 kPa. The predicted bundle elastic modulus would be 79 kPa or 59 kPa had the ECM percentage been changed to 6% or 4%, respectively.

**Figure 5:**
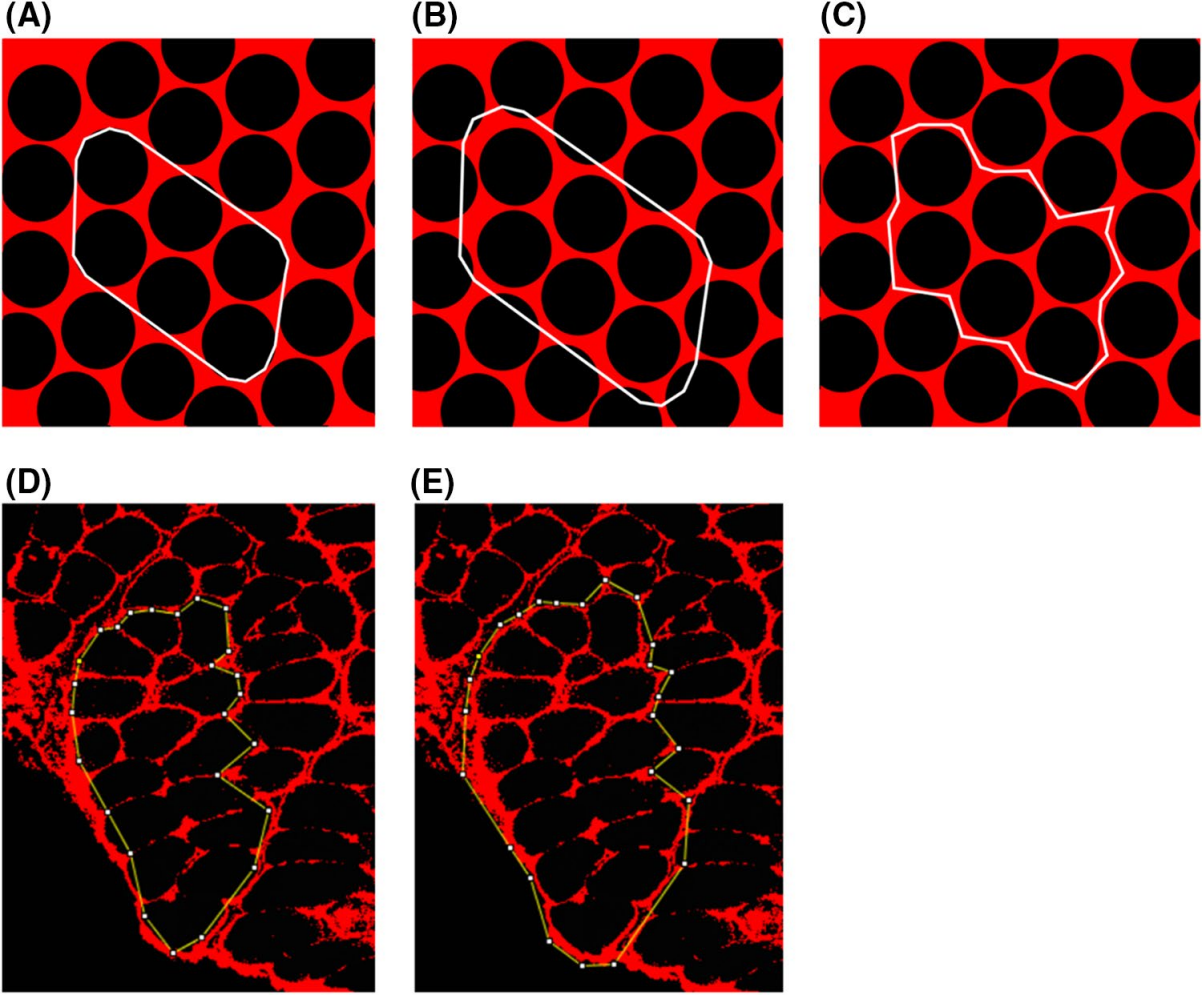
Segmentation of fiber bundles from immunohistochemistry images. Two segmentations were performed for each bundle: the borders of one segmentation (type A) passed internal to the edge of boundary fibers of the bundle (**A**,**D**), while borders of the other segmentation (type B) passed externally, including the boundary edge of the fibers immediately adjacent but external to the bundle (**B**,**E**). Schematic representations for a simulated bundle of 6 fibers, are shown for segmentation type A (**A**), segmentation type B (**B**), and how a real bundle may present (**C**). The collagen I deposition was measured for the two segmentations (**D**,**E**) and their average was considered for the selected bundle.

To further explore this idea, we performed an immunostaining analysis to quantify ECM content in bundles of different sizes from multifidus of one rat in G1 as an example. While the exact relationship between ECM elastic modulus and its constituents is not yet clear, many studies report collagen I as the major contributor to elastic modulus of ECM^15,29^. Therefore, we measured and contrasted collagen I content in bundles of different sizes. A portion of the collected biopsy was separated and immediately snap frozen in isopentane cooled by liquid nitrogen. The biopsy was sectioned, placed on slides, and immune-stained for collagen I. Using Image J software, bundles of different sizes were arbitrarily defined, segmented and their collagen I area fraction (i.e. the ratio of collagen I area over the entire bundle cross sectional area) was measured. As no collagen I exists inside muscle fibers, the number of fibers within each bundle can easily be counted. However, as the exact border of bundles cannot be determined from the images, two segmentations (types A and B) were performed for each bundle: the borders of one segmentation (type A) passed internal to the edge of boundary fibers of the bundle (Fig. 5A,D), while borders of the other segmentation (type B) passed externally, including the boundary edge of the fibers immediately adjacent but external to the bundle (Fig. 5B,E). The collagen I deposition was measured for these two segmentations and their average was considered for the selected bundle.

**Figure 6:**
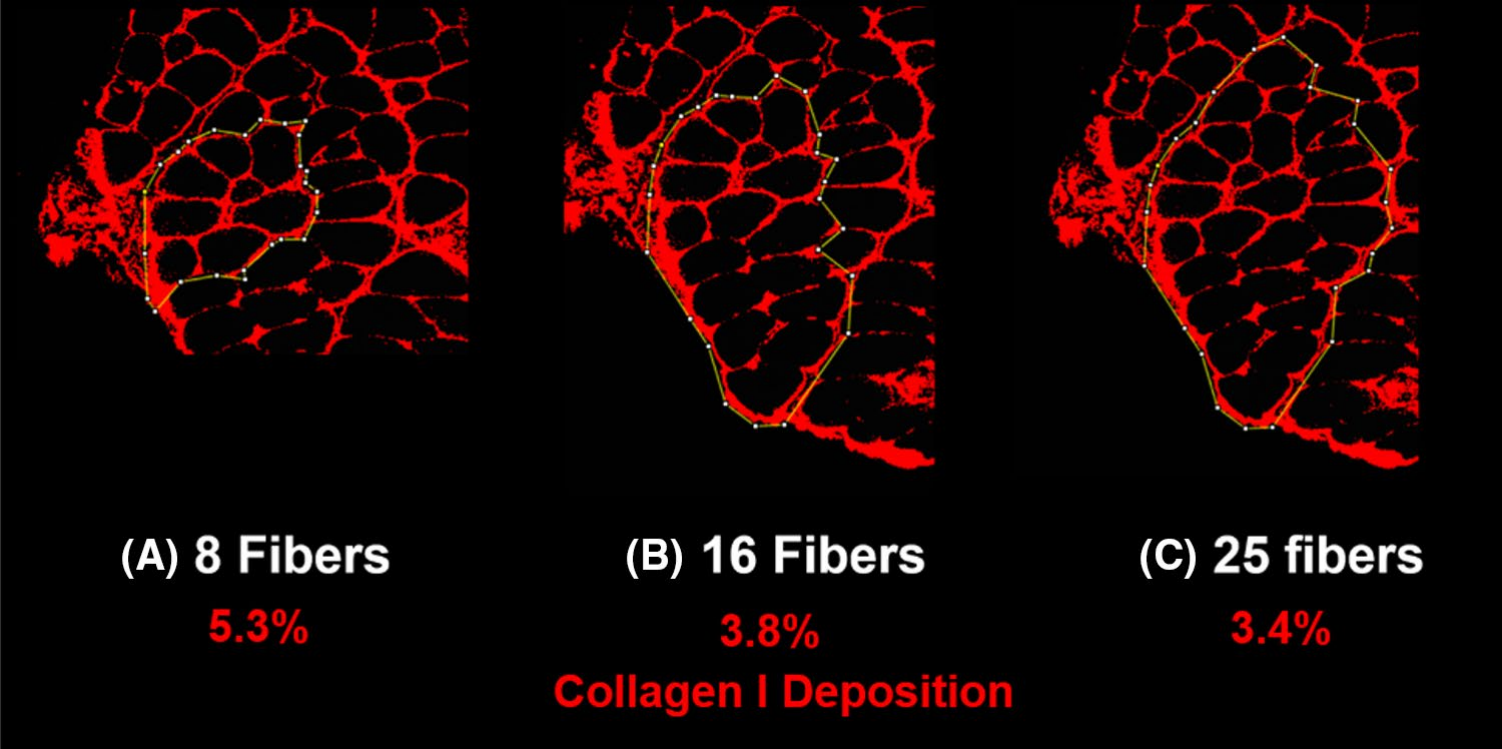
Inverse correlation between collagen I deposition and size of three bundles of different sizes from multifidus of one rat in G1. The measured percentage of collagen I deposition for bundles A, B, and C were 5.3%, 3.8%, and 3.4%, respectively.

Following this approach, collagen I deposition of larger bundles was measured to be smaller for the studied biopsy (Fig. 6). The three bundles studied had 8, 16, and 25 fibers but their collagen I content was 5.3%, 3.8%, and 3.4% of the area, respectively. Assuming an elastic modulus of ~ 20 kPa for muscle fibers and ~ 1 MPa for the ECM^28^ and using the rule of mixture, the corresponding elastic moduli of these three bundles are estimated as 72, 57, and 53 kPa, respectively.

**Figure 7:**
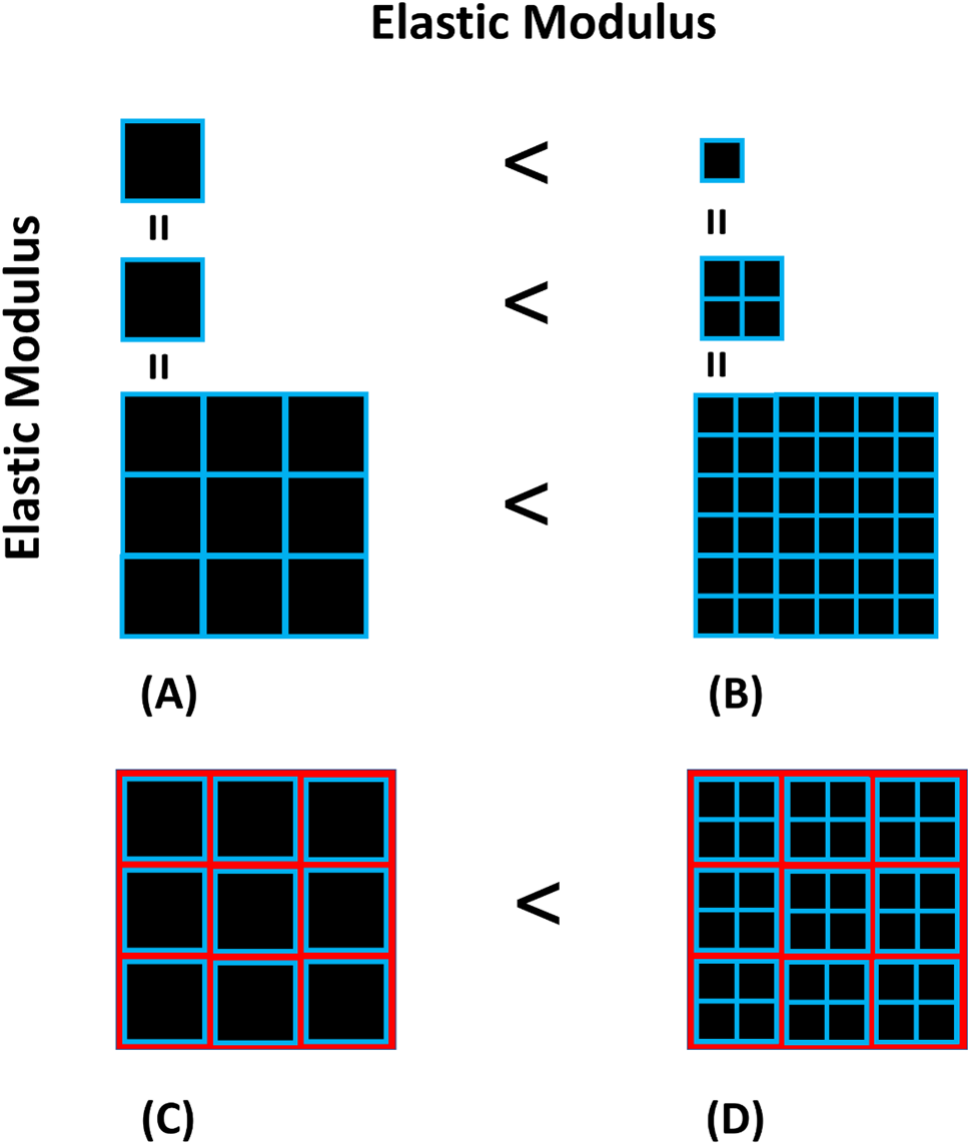
Effect of fiber size on bundle elastic modulus. In all images, black color represents the contractile elements of muscle fibers, blue color represents basement membranes of muscle fibers, and red color represents the ECM. Given that larger fibers have smaller elastic moduli (**A**,**B**), for two muscle bundles with same ECM content (**C**,**D**), the one having more fibers (i.e. consisting of smaller fibers) will have a higher elastic modulus.

Another factor contributing to the elastic modulus of a bundle could be the distribution of fiber sizes within that bundle. As our results suggest, larger fibers have a smaller modulus. Therefore, for two muscle bundles with same amount of ECM, the one having more fibers (i.e. consisting of smaller fibers) will have a higher elastic modulus (Fig. 7).

While both factors, i.e. collagen I deposition and fiber size distribution, could potentially explain the results of our study, further investigation is required to find out their relative effects. Although we reasonably used the average of type A and B segmentations for our sample measurement of collagen I percentage, the actual bundle that was mechanically tested might have a different collagen I content (Fig. 5C) that could be closer to either segmentation A or B. Notably, for the three bundles in Fig. 6, segmentation type A would result in larger percentages for larger bundles, while type B segmentation and the average method showed an opposite trend. Therefore, it is quite important to know how much ECM remains on an actual extracted bundle, for example by measuring the volumetric collagen deposition of a bundle using a three dimensional imaging technique or hydroxyproline assay^29^.

It is noteworthy that if the larger fibers/bundles manifest shorter slack sarcomere lengths, because of how we used a set force threshold to determine this length, then 30% strain would occur at shorter absolute sarcomere lengths which could influence the calculated moduli. To explore whether the smaller elastic modulus of larger bundles was due to differences in their slack sarcomere lengths we fitted a linear regression model to identify any relationship between the slack sarcomere lengths and CSAs of the tested bundles. No significant linear correlation was found for any of the groups except for G1 longissimus fibers (P < 0.05). This confirmed that the observed difference in elastic moduli of the tested bundles with varying sizes was not an artifact of the slack sarcomere length measurement method.

The linear regression approach in the current study revealed a statistically significant effect of CSA on the elastic modulus of all fibers and fiber bundles (except for G3 multifidus fibers and G2 longissimus bundles). That the ${R}^{2}$ values for this association were relatively low (all ${R}^{2}<0.30$) is not surprising. The low values for the ${R}^{2}$ could stem from the biological variations in the subjects or from the variations in sites within biopsies from where fibers and fiber bundles were extracted; thus suggesting possible role of other factors that were not studied here. What is noteworthy is the found dependence of elastic modulus on CSA despite being already normalized by the CSA, which means that the size of the fibers and fiber bundles needs to be considered.

For the second objective of this study, the intriguing observation was that muscle fibers and fiber bundles were not cylindrical (all P < 0.0001). In contrast to the shape of a single muscle fiber, the shape of a muscle fiber bundle could be somewhat controlled by the person extracting the bundle from the biopsy tissue. However, during extraction to maintain the integrity of a bundle, it may be necessary to avoid separating extra fibers that can result in loss of bundle integrity. This will result in bundle shapes that may not be cylindrical. Many studies only measure fiber and fiber bundle diameter along a single axis typically from the top view^14,15,18–21^. The cross section of fibers and fiber bundles is then assumed to possess a cylindrical shape. However, measurement of diameters from two orthogonal axes (top and side view) in the current study revealed that such an assumption was not valid in rodents or humans, especially at the bundle level. The ratio of major axis over the minor axis of a cylindrical sample should be equal to 1, whereas the median for ratio of major axis over the minor axis of the samples tested in this study ranged between 1.15 and 1.29 for fibers and 1.27 and 1.44 for fiber bundles. The implication of this finding is that the measurement of elastic modulus values could be off by a factor of 1.15 to 1.29 for fibers and 1.27 to 1.44 for fiber bundles if the diameter along only one axis is measured. Therefore, it is recommended to measure the fiber and bundle diameters from both top and side views.

Muscle is an organized material with several structural levels. In a recent study, Ward et al.^15^ measured the elastic modulus of rabbit muscles at multiple levels, i.e. single fibers, fiber bundles (~ 20 fibers), fascicles (~ 300 fibers), and whole muscles. They found that elastic modulus increases nonlinearly with these size scales as does the collagen content. The results of the current study demonstrated that larger bundles were associated with lower elastic moduli. These results are not in conflict, but rather they are complementary. Our results suggest that for fiber bundles of less than ~ 50 fibers larger sizes will be associated with smaller elastic moduli. However, beyond a certain size (e.g. ~ 300 fibers), bundles will transition to true fascicles, including the presence of perimysium and higher amounts of collagenous tissue, thereby leading to larger elastic moduli compared to bundles. In conclusion, the findings of our study suggest that similar size of bundles should be tested when comparing for differences between groups.

## Supplementary Information

Supplementary Information.

## Data Availability

All data generated or analyzed during this study are included in figures of the current manuscript.
