# Supplementary material for: Larger muscle fibers and fiber bundles manifest smaller elastic modulus in paraspinal muscles of rats and humans
Source: Sci Rep. 2021 Sep 17;11:18565. doi: 10.1038/s41598-021-97895-z (PMC8448745; doi:10.1038/s41598-021-97895-z)
Supplement: Supplementary file 1 — Supplementary Information. [file 41598_2021_97895_MOESM1_ESM.docx]

| Table S1. Patient Demographics. All patients recruited for this study were operated at Vancouver General Hospital. | | | | | | |
| --- | --- | --- | --- | --- | --- | --- |
|  |  |  |  |  | **Coronal Deformity** | |
| **Ptnt**  **#** | **Gender** | **Age**  **(yrs)** | **Diagnosis** | **Levels**  **Affected** | **Severity (Cobb Angle)** | **Apex (Two-Ends)** |
| 1 | F | 70 | Scoliosis, degenerative, Second, acquired | T10-L4 | Severe (58°) | L1 (T11-L3) |
| 2 | M | 64 | Spinal stenosis | L4-S1 | Moderate (30°) | L2 (L1-L3) |
| 3 | M | 61 | Spinal stenosis | L4-L5 | Very Mild (8°) | L2 (T12-L4) |
| 4 | M | 75 | Spondylosis | L2-S1 | Mild (17°) | L2 (L2-L4) |
| 5 | M | 71 | Spinal stenosis | L2-L4 | Mild (13°) | T12-L1 (T10-L2) |
| 6 | F | 59 | Spinal stenosis | L5-S1 | Mild (16°) | L3 (L2-L4) |
| 7 | F | 70 | Spinal stenosis | L2-S1 | Very Mild (4°) | L2 (T12-L4) |
| 8 | M | 73 | Spinal stenosis | L3-L5 | None (0°) | None |
| 9 | F | 51 | Scoliosis, degenerative, Second, acquired | T9-S2  &ILIUM | Severe (57°) | L2 (T12-L3) |
